# Liver proteomics unravel the metabolic pathways related to Feed Efficiency in beef cattle

**DOI:** 10.1038/s41598-019-41813-x

**Published:** 2019-03-29

**Authors:** Leydiana D. Fonseca, Joanir P. Eler, Mikaele A. Pereira, Alessandra F. Rosa, Pâmela A. Alexandre, Cristina T. Moncau, Fernanda Salvato, Livia Rosa-Fernandes, Giuseppe Palmisano, José B. S. Ferraz, Heidge Fukumasu

**Affiliations:** 1 Department of Veterinary Medicine, School of Animal Science and Food Engineering, University of São Paulo, Pirassununga, 13635-900 Brazil; 2 Institute of Biology, State University of Campinas, Campinas, 13083-862 Brazil; 3 Department of Parasitology, Biomedical Sciences Institute, University of São Paulo, São Paulo, 05508-900 Brazil

## Abstract

Improving nutrient utilization efficiency is essential for livestock, given the current scenario of increasing demand for animal protein and sustainable resource use. In this context, understanding the biology of feed efficiency (FE) in beef cattle allows the development of markers for identification and selection of best animals for animal production. Thus, 98 young Nellore bulls were evaluated for FE and at the end of the experiment liver samples from six High Feed Efficient (HFE) and six Low Feed Efficient (LFE) animals were collected for protein extraction, digestion and analysis by HPLC-MS/MS. Data were analyzed for differential abundant proteins (DAPs), protein networks, and functional enrichment. Serum endotoxin was also quantified. We found 42 DAPs and 3 protein networks significantly related to FE. The main pathways associated with FE were: microbial metabolism; biosynthesis of fatty acids, amino acids and vitamins; glycolysis/gluconeogenesis; xenobiotic metabolism and; antigen processing and presentation. Serum endotoxins were significantly higher in LFE animals supporting the results. Therefore, the findings presented here confirmed the altered hepatic metabolism and pronounced hepatic inflammation in LFE animals supporting that the increased bacterial load is at least in part responsible for the hepatic lesions and inflammation in LFE animals.

## Introduction

Improving nutrient utilization efficiency is essential for the viability of animal production, given the current scenario of increasing demand for animal protein and the needs of sustainable resource use. In this context, understanding the biology of feed efficiency (FE) in beef cattle will allow the development of markers for identification and selection of best animals for animal production. Complex biological processes are related with the utilization of feed by the animals^1^, varying due to many factors like amount and type of feed consumed, sex, breed and environmental aspects^2^. In the last years, several studies have analyzed the genetic and molecular mechanisms that are associated with feed efficiency (FE) in animals. The identification of genomic regions, candidate genes and single nucleotide polymorphisms (SNPs) potentially associated with FE in cattle has been the focus for both taurine^3–9^ and zebu cattle, especially Nellore breed^10–15^, being the metabolism of lipids, proteins and energy, immune response, signaling pathways, and ions transport frequently associated with FE in these studies.

The analysis of gene expression by transcriptomic methods has also been widely diffused, with several target tissues, relating differences in expression and networks of gene interaction to FE^16–24^. The knowledge of the gene expression on different conditions assists in the understanding regarding the function of the gene and regulation routes^25^. However, there is not always a relationship between gene expression and abundance of proteins, the main functional structures of the cell, which suggests distinct mechanisms of control at these levels^25,26^. Thus, it is interesting to determine whether the pathways and biological processes associated with FE at the mRNA level would also be relevant at the protein level. Even with all potential application in animal production and health, proteomic approach is still little explored in production animals, being limited mainly by costs, lack of good genomic data for species of interest, as well as lack of awareness of the potentialities of this tool^27^.

The liver, in turn, is a complex organ that plays a fundamental function for many essential metabolic processes, besides being the central organ in the energy metabolism. Liver proteomes analyzes allow, in addition to a better understanding of the function of the organ itself, a greater knowledge of the biochemical and physiological aspects of animal metabolism as a whole^28^. In this way, the study of the liver by proteomic approach can broaden our current knowledge about the molecular mechanisms determining FE and help in the search for biomarkers to identify efficient animals early and optimize beef cattle production. In this sense, the present work compared the proteomic profile and analyzed the co-expression networks of proteins identified in the liver of Nellore cattle with high feed efficiency (HFE) and low feed efficiency (LFE).

## Results

### Phenotypic data

A summary of the phenotypic data from the animal experiment is found in Table 1 (the complete phenotypic data could be found in Alexandre *et al*.^16^).

**Table 1:** Descriptive statistics of high feed efficiency (HFE) and low feed efficiency (LFE) for phenotypic traits.

Trait	HFE (±SEM)	LFE (±SEM)	p-value
BWi (kg)	403.10 ± 35.6	409.50 ± 23.0	0.5
BWf (kg)	542.10 ± 46.9	533.90 ± 25.2	0.47
DMI (kg/d)	9.99 ± 1.3	12.00 ± 0.7	1.03 × 10^−6^*
ADG (kg/d)	1.97 ± 0.5	1.76 ± 0.2	0.06
FCR	5.22 ± 0.8	6.90 ± 0.8	2.84 × 10^−8^*
RFI (kg/d)	−1.14 ± 0.4	1.24 ± 0.5	6.79 × 10^−8^*
RWG (kg/d)	0.27 ± 0.3	−0.29 ± 0.2	3.00 × 10^−9^*
RIG	1.40 ± 0.4	−1.53 ± 0.6	6.77 × 10^−8^*
REAi (cm^2^)	67.51 ± 5.5	65.95 ± 5.3	0.36
REAf (cm^2^)	83.49 ± 6.9	83.12 ± 6.0	0.85
REAg (cm^2^)	15.98 ± 8.9	17.18 ± 6.4	0.63
BFTi (mm)	1.18 ± 1.0	1.64 ± 1.2	0.19
BFTf (mm)	3.99 ± 1.9	5.78 ± 1.4	1.9 × 10−3*
BFTg (mm)	2.81 ± 2.0	4.15 ± 1.4	0.02*

BWi, initial body weight; BWF, final body weight; DMI, dry matter intake; ADG, average daily gain; FCR, feed conversion ratio; RFI, residual feed intake; RWG, residual body weight gain; RIG, residual intake and body weight gain; REAi, initial rib eye area; REAf, final rib eye area; REAg, gain of rib eye area; BFTi, initial back fat thickness; BFTf, final back fat thickness; BFTg, gain of back fat thickness; RFTi, initial rump fat thickness; RFTf, final rump fat thickness; RFTg, gain of rump fat thickness; LW, liver weight; CY, carcass yield; PFW, pelvic fat weight; KFW, kidney fat weight. *P ≤ 0.05. Adapted from Alexandre *et al*.^16^.

### Differential proteomic analysis

**Figure 1:**
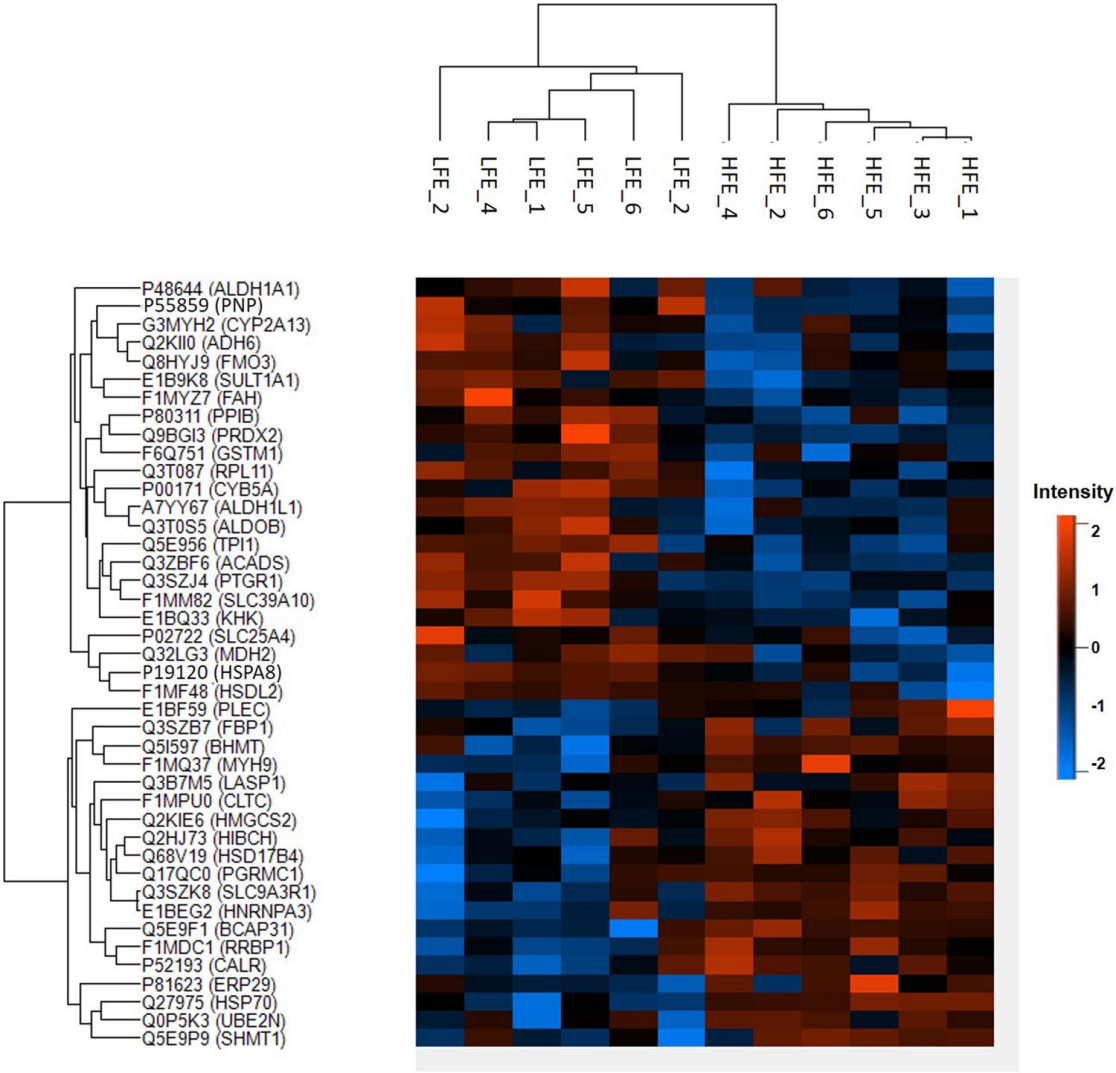
Dendrogram representing the hierarchical cluster of differentially abundant proteins among liver samples from Nellore cattle classified as high and low feed efficiency. HFE: high feed efficiency; LFE: low feed efficiency.

Summary information about mass spectrometry analysis can be found at Supplementary Table S1. A total of 529 proteins were identified in this work of which 376 proteins were maintained after the use of exclusion criteria (contaminant proteins, identification of reverse database and presence in at least 50% of the samples from both experimental groups, Supplementary Table S2). From these, 42 were statistically different between HFE and LFE groups, of which 23 proteins were more abundant in the LFE group (Supplementary Table S3). Cluster hierarchization using the differentially abundant proteins (DAPs) confirmed the presence of two distinct groups (Fig. 1).

### Interaction network and functional enrichment of differentially abundant proteins

**Figure 2:**
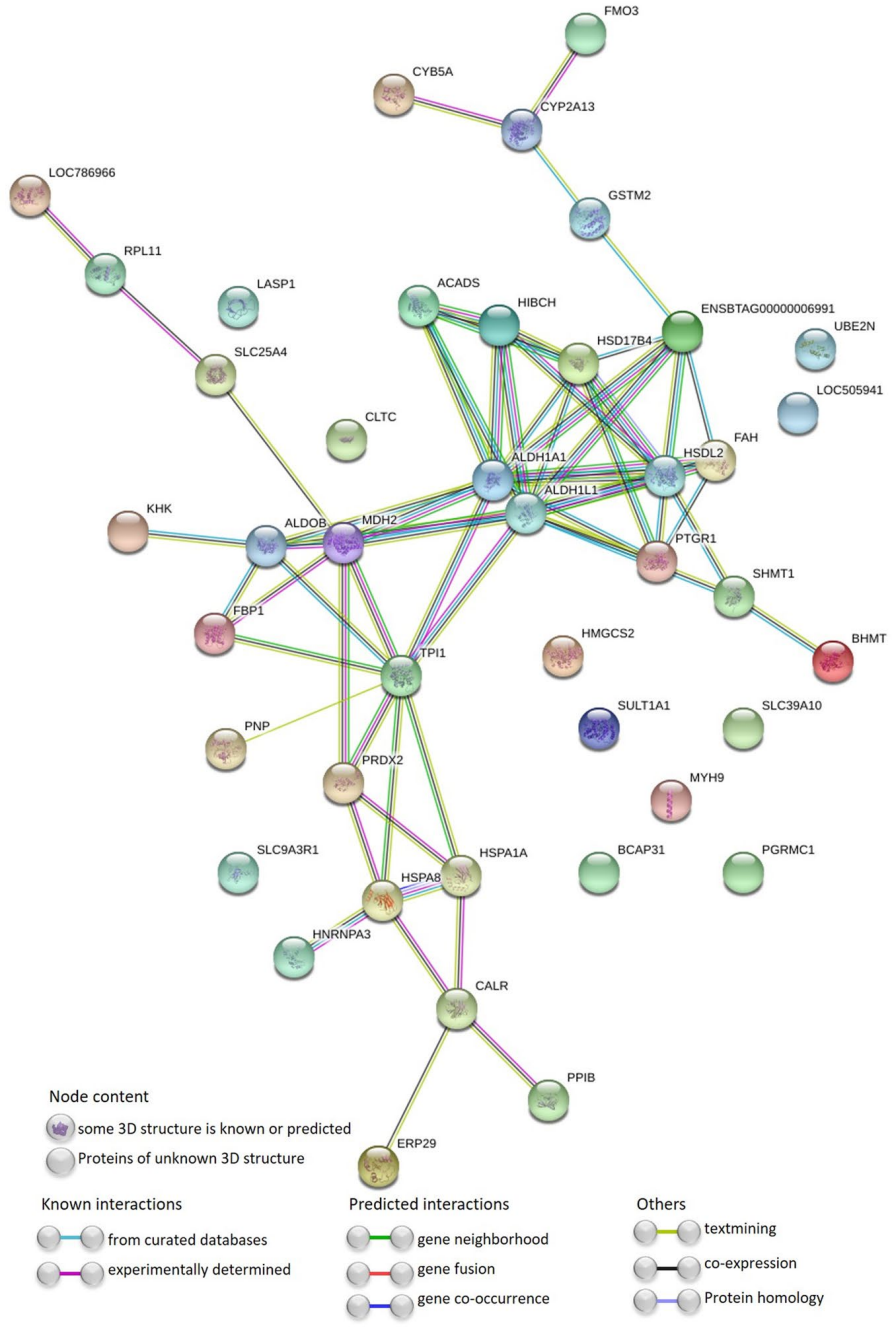
Protein-protein interaction network of differentially abundant proteins in the liver of Nellore cattle classified to feed efficiency. Nodes represent the differentially abundant proteins that are identified with the coding gene symbol. Colored nodes indicate the query proteins. Lines represent the connections between proteins.

The interaction network between the DAPs was highly significant (p-value < 10^−16^), indicating that the DAPs are at least partially biologically connected (Fig. 2). Only 11 proteins did not present interactions. Seventeen metabolic pathways were enriched (p-adjust < 0.05) from this network (Table 2), with interesting metabolic pathways as *microbial metabolism in diverse environments*, *glycolysis/gluconeogenesis*, *drug metabolism - cytochrome P450*, *antigen processing and presentation*, etc.

**Table 2:** Enriched metabolic pathways of differentially abundant hepatic proteins among low and high feed efficiency Nellore cattle.

KEGG ID	Description	p-value	p-adjust	Genes	PN
01120	*Microbial metabolism in diverse environments*	7.11 × 10^−10^	2.02 × 10^−07^	**MDH2**; **ALDOB**; **FMO3**; SHMT1; FBP1; **FAH**; **SULT1A1**; **TPI1**	8
01100	*Metabolic pathways*	5.34 × 10^−08^	5.77 × 10^−06^	**PNP**; **FAH**; BHMT; **ALDOB**; SHMT1; **MDH2**; FBP1; **ADH6**; HSD17B4; **ALDH1A1**; **KHK**; **ACADS**; **TPI1**; HMGCS2	14
01200	*Carbon metabolism*	6.10 × 10^−08^	5.77 × 10^−06^	**ACADS**; **MDH2**; **ALDOB**; SHMT1; FBP1; **TPI1**	6
00051	*Fructose and mannose metabolism*	5.08 × 10^−07^	3.61 × 10^−05^	**ALDOB**; FBP1; **TPI1**; **KHK**	4
04141	*Protein processing in endoplasmic reticulum*	1.10 × 10^−06^	6.26 × 10^−05^	ERP29; BCAP31; CALR; **HSPA8**; HSPA1A; RRBP1	6
00010	*Glycolysis/Gluconeogenesis*	6.57 × 10^−06^	3.11 × 10^−04^	**ALDOB**; **ADH6**; FBP1; **TPI1**	4
05204	*Chemical carcinogenesis*	8.05 × 10^−06^	3.26 × 10^−04^	**ADH6**; **LOC540707**; **SULT1A1**; **GSTM2**	4
00982	*Drug metabolism - cytochrome P450*	1.58 × 10^−04^	5.60 × 10^−03^	**ADH6**; **FMO3**; **GSTM2**	3
00980	*Metabolism of xenobiotics by cytochrome P450*	1.77 × 10^−04^	5.60 × 10^−03^	ADH6; LOC540707; GSTM2	3
04612	*Antigen processing and presentation*	3.76 × 10^−04^	1.07 × 10^−02^	CALR; **HSPA8**; HSPA1A	3
01230	*Biosynthesis of amino acids*	4.28 × 10^−04^	1.10 × 10^−02^	**ALDOB**; SHMT1; **TPI1**	3
00670	*One carbon pool by folate*	6.45 × 10^−04^	1.53 × 10^−02^	SHMT1; **ALDH1L1**	2
00630	*Glyoxylate and dicarboxylate metabolism*	1.15 × 10^−03^	2.52 × 10^−02^	SHMT1; **MDH2**	2
00650	*Butanoate metabolism*	1.25 × 10^−03^	2.54 × 10^−02^	**ACADS**; HMGCS2	2
00030	*Pentose phosphate pathway*	1.36 × 10^−03^	2.57 × 10^−02^	**ALDOB**; FBP1	2
00270	*Cysteine and methionine metabolism*	2.32 × 10^−03^	4.11 × 10^−02^	BHMT; **MDH2**	2
00350	*Tyrosine metabolism*	2.89 × 10^−03^	4.82 × 10^−02^	**ADH6**; **FAH**	2

p-adjust: p-value corrected to multiple tests by FDR (Benjamini-Hochberg); PN: protein number in the pathway. Font bold genes correspond to the most abundant proteins in the low feed efficiency group.

**Figure 3:**
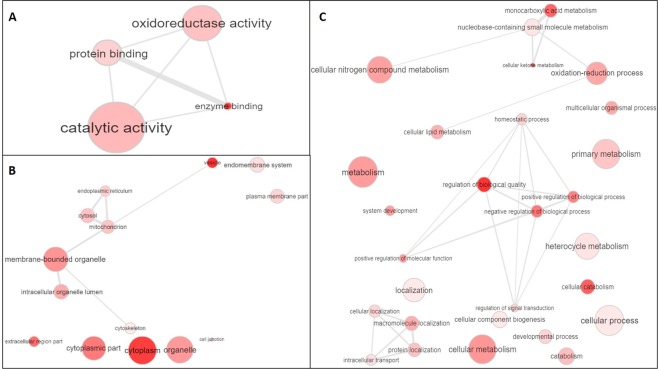
Enriched terms (p-adjusted < 0.05, corrected by Bonferroni method) for molecular function (**A**) and, after semantic synthesis by Revigo, for cellular component (**B**) and biological process (**C**) of differentially abundant liver proteins among Nellore cattle classified to feed efficiency. The intensity of color circles is proportional to the significance of the term while the size is proportional to the number of proteins identified in each term.

The DAPs were also enriched for GO identifying 67 significant terms (p-adjust < 0.05). Four of these terms corresponded to molecular function (Fig. 3A). Most of the proteins identified the *catalytic activity* (GO:0003824) and *oxidoreductase activity* (GO:0016491) terms in the LFE, whereas *protein binding* (GO: 0005515) was less abundant in this group (Supplementary Table S4). For cellular components, 22 terms were enriched, remaining 14 terms after semantic synthesis (Fig. 3B). The five most significant non-redundant terms were *vesicle* (GO:0031982), *cytoplasm* (GO:0005737), *extracellular region part* (GO:0044421), *cytoplasmic part* (GO:0044444), and *membrane-bounded organelle* (GO:0043227) (Supplementary Table S5). The biological process ontology presented 41 enriched terms (p-adjust < 0.05), leaving 28 terms after semantic synthesis (Fig. 3C). The five most significant non-redundant processes were *regulation of biological quality* (GO:0065008), *cellular ketone metabolic process* (GO:0019752), *cellular catabolic process* (GO:0044248), *monocarboxylic acid metabolic process* (GO:0032787), and *negative regulation of biological process* (GO:0048519) (Supplementary Table S6).

### Co-expression network and enrichment pathways of modules correlated to feed efficiency

**Figure 4:**
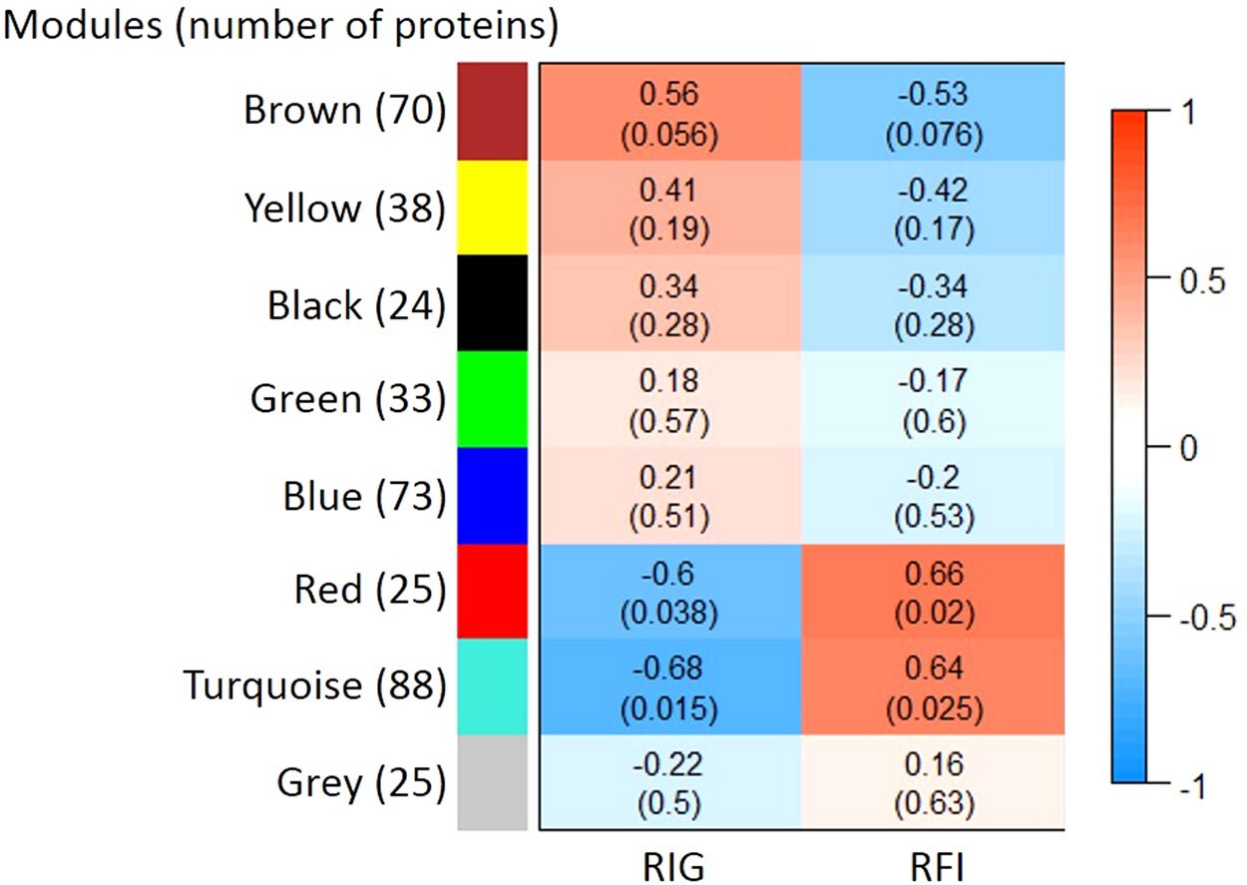
Correlations between hepatic proteins co-expression networks and feed efficiency traits of Nellore cattle. RIG: residual intake and body weight gain; RFI: residual feed intake. Modules represent the network of co-expressed proteins and are named by different colors. Correlations are presented in the rectangles followed by the p-value in parentheses.

From the co-expression analysis by WGCNA, the 376 quantified proteins were clustered based on their expression patterns into eight modules, that were named by different colors (Fig. 4). The gray module, in turn, does not represent a network of co-expression. In this module are grouped those proteins that did not fit any other module. According to Fig. 4, three modules were correlated with FE traits (p < 0.10). The brown module was positively associated to residual intake and body weight gain (RIG) and negatively to residual feed intake (RFI), thus being related to higher efficiency, while the turquoise and the red modules were associated with lower efficiency. Of the 42 DAPs between LFE and HFE groups, only seven proteins were not present in the significant modules (Supplementary Table S7). A network with the proteins present in the three significant modules can be visualized in the Supplementary Fig. S1.

Turquoise module showed 88 proteins of which 18 proteins were most abundant in LFE group. Of the total, 34 were strongly affiliated to the module, with module membership (MM) values > 0.60 and p-value < 0.05 (Supplementary Table S8). The red module contained 25 proteins, and 17 of these presented MM > 0.60 (p-value < 0.05) (Supplementary Table S9). Five DAPs were distributed in this module and only *Purine nucleoside phosphorylase* (PNP) showed MM below the determined limit (MM = 0.58; p-value = 0.047). Brown module had 70 proteins, and 32 of these with high MM values (>0.60 and p-value < 0.05) (Supplementary Table S10). Twelve proteins in this module were DAPs and 10 of these presented higher MM values, all more abundant in HFE. The two DAPs with MM below the established minimum were *B-cell receptor-associated protein 31* (BCAP31) (MM = 0.48; p-value = 0.11) and *Serine hydroxymethyltransferase*, *cytosolic* (SHMT1) (MM = 0.31; p-value = 0.33).

The functional enrichment analysis for the three modules significantly associated with FE is presented in Table 3. The turquoise module associated to LFE identified 11 significant pathways (p-adjust < 0.05) being nine common to pathways enriched previously for DAPs, as *Microbial metabolism in diverse environments* and *Drug metabolism - cytochrome P450*. The red module, also associated with LFE, enriched for seven metabolic pathways being all common to the pathways obtained for DAPs, except for the *Retinol metabolism*. At last, the brown module (associated with HFE) enriched for five metabolic pathways being three in common to more expressed DAPs in HFE animals.

**Table 3:** Enriched metabolic pathways of hepatic proteins of Nellore cattle co-expressed in the modules significantly correlated to feed efficiency traits.

KEGG ID	Description	p-value	p-adjust	Genes	PN
**Turquoise module**					
01100	*Metabolic pathways*	1.36 × 10^−09^	3.85 × 10^−07^	**FAH**; **ALDOB**; DPYS; HADHB; PSAT1; COX5A; MTHFD1; **ADH6**; ACADL; ADH4; EPHX2; **KHK**; **ACADS**; **TPI1**	14
01120	*Microbial metabolism in diverse environments*	3.84 × 10^−09^	5.45 × 10^−07^	PSAT1; **ALDOB**; **FMO3**; **FAH**; EPHX2; **TPI1**; HADHB	7
00071	*Fatty acid degradation*	7.27 × 10^−09^	6.88 × 10^−07^	ACADL; **ADH6**; ADH4; **ACADS**; HADHB	5
00010	*Glycolysis/Gluconeogenesis*	2.45 × 10^−06^	1.74 × 10^−04^	**ALDOB**; **ADH6**; ADH4; **TPI1**	4
00051	*Fructose and mannose metabolism*	1.78 × 10^−05^	1.01 × 10^−03^	**ALDOB**; **TPI1**; **KHK**	3
01200	*Carbon metabolism*	2.16 × 10^−05^	1.02 × 10^−03^	PSAT1; **ACADS**; **ALDOB**; **TPI1**	4
00350	*Tyrosine metabolism*	3.32 × 10^−05^	1.35 × 10^−03^	**ADH6**; ADH4; **FAH**	3
01212	*Fatty acid metabolism*	6.74 × 10^−05^	2.39 × 10^−03^	**ACADS**; ACADL; HADHB	3
00982	*Drug metabolism - cytochrome P450*	7.62 × 10^−05^	2.40 × 10^−03^	**ADH6**; **FMO3**; ADH4	3
01230	*Biosynthesis of amino acids*	2.08 × 10^−04^	5.91 × 10^−03^	**ALDOB**; PSAT1; **TPI1**	3
00670	*One carbon pool by folate*	3.98 × 10^−04^	1.03 × 10^−02^	MTHFD1; **ALDH1L1**	2
**Red module**					
01100	*Metabolic pathways*	1.94 × 10^−06^	5.51 × 10^−04^	**MDH2**; AOX1; ADH1C; CKB; RGN; ADK; **ALDH1A1**; TKT	8
01120	*Microbial metabolism in diverse environments*	6.37 × 10^−06^	9.04 × 10^−04^	RGN; **MDH2**; AOX1; TKT	4
00830	*Retinol metabolism*	1.16 × 10^−05^	1.10 × 10^−03^	**ALDH1A1**; ADH1C; AOX1	3
01200	*Carbon metabolism*	7.85 × 10^−05^	5.57 × 10^−03^	RGN; **MDH2**; TKT	3
00030	*Pentose phosphate pathway*	2.19 × 10^−04^	1.24 × 10^−02^	RGN; TKT	2
00350	*Tyrosine metabolism*	4.70 × 10^−04^	2.22 × 10^−02^	ADH1C; AOX1	2
00982	*Drug metabolism - cytochrome P450*	8.14 × 10^−04^	3.30 × 10^−02^	ADH1C; AOX1	2
**Brown module**					
01100	*Metabolic pathways*	8.48 × 10^−06^	2.41 × 10^−03^	ACSM2A; GRHPR; ACY1; LAP3; RDH16; PGM2; DHRS4; **HSD17B4**; LDHB; **HMGCS2**	10
00480	*Glutathione metabolism*	6.94 × 10^−05^	9.85 × 10^−03^	GSTA5; GSTA1; LAP3	3
04141	*Protein processing in endoplasmic reticulum*	1.28 × 10^−04^	1.21 × 10^−02^	PDIA3; **CALR**; **RRBP1**; HSP90B1	4
04918	*Thyroid hormone synthesis*	1.74 × 10^−04^	1.23 × 10^−02^	ASGR1; ATP1A2; HSP90B1	3
00650	*Butanoate metabolism*	7.28 × 10^−04^	4.13 × 10^−02^	ACSM2A; **HMGCS2**	2

p-adjust: p-value corrected to multiple tests by FDR (Benjamini-Hochberg); PN: protein number in the pathway; Modules correspond to co-expression networks and are named by different colors. Turquoise and red modules are associated with low feed efficiency and brown module is associated with high feed efficiency. Font bold genes correspond to differentially abundant proteins between low and high feed efficiency groups.

### Serum endotoxin quantification

**Figure 5:**
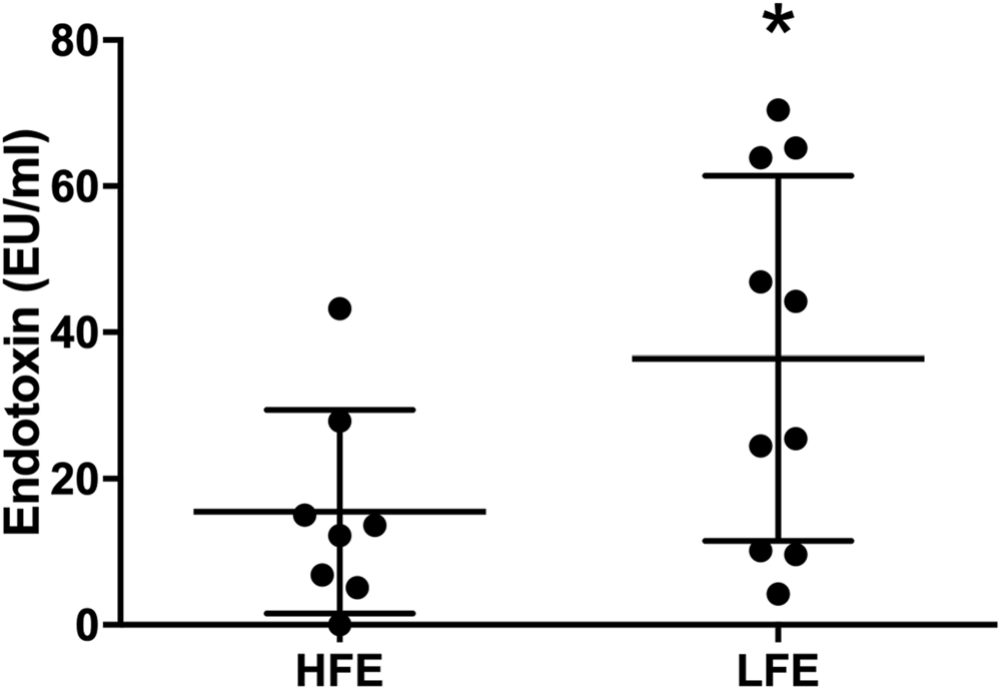
Quantification of serum endotoxin from high and low feed efficiency animals. HFE: high feed efficiency; LFE: low feed efficiency; *P ≤ 0.05.

To validate the most significant pathway related to LFE (*microbial metabolism in diverse environments*) in this experiment, serum endotoxin was measured. The LFE group had significantly more serum endotoxin than the HFE group corroborating the proteomic results (HFE:15.48 ± 4.93 EU/mL, n = 8 and LFE: 36.46 ± 7.90 EU/mL, n = 10; P ≤ 0.05, Fig. 5).

## Discussion

In this study, we used liver proteomics to unravel potential metabolic pathways related to feed efficiency in beef cattle. Comparison between hepatic proteomes of high and low FE animals revealed 42 proteins significantly different and three networks of co-expressed proteins (named turquoise, red and brown modules) significantly associated to FE. The functional enrichment of these data indicated metabolic pathways as microbial metabolism in diverse environments, biosynthesis of fatty acids, amino acids, vitamins and hormones, glycolysis/gluconeogenesis, cytochrome p450 and antigen processing and presentation as the most important metabolic pathways for feed efficiency in beef cattle in our conditions. Part of these results are in accordance with our previous work based on transcriptomic analysis^16^ and from others^17–24^ which strengths the importance of the findings presented in this work. It is worth highlighting that in a recent study using the proteomic approach based on two-dimensional electrophoresis in combination with mass spectrometry (ESI-MS) to investigate alterations in the liver of Nellore cattle divergently ranked to RFI the authors identified proteins related to oxygen transport and blood flow; mitochondrial function and energy metabolism; amino acid metabolism, ion transport, and cell survival^29^.

In our previous study with the same animals analyzed here, there was no difference in body weight gain between LFE and HFE groups (Table 1), but higher dry matter intake during the whole experiment and an increase in serum gamma-glutamyl transpeptidase (GGT) at the end of the experiment was noted for LFE groups. There was also higher deposition of both visceral and subcutaneous fat and greater expression of genes associated with lipid synthesis in the LFE group^16^. In addition, animals from both groups presented mononuclear infiltrate in the portal triad, but at higher levels in LFE animals^16^. Based on these observations, we hypothesized that less feed efficient animals presented more hepatic lesions because of the stress produced by altered lipid metabolism and/or due to increased bacterial infection due to higher feed intake^16^. Thus, the results of proteomic approach of the present study corroborated these findings since the term *Microbial metabolism in diverse environments* was the most enriched pathway for DAPs, with predominance of more abundant proteins in LFE, and the second most enriched route for two co-expressed networks (turquoise and red modules). In addition, we demonstrated that LFE animals have significantly more serum endotoxins than HFE animals suggesting increased bacterial load in LFE animals. The proteomics and serum endotoxin analyzes presented here strongly support the microorganisms as at least one of the causes for increased hepatic lesions and consequently more inflammation in LFE as previously described Alexandre *et al*.^16^. Another relevant term to reinforce this results was *antigen processing and presentation* that was also associated with FE in previous studies^5,30,31^. The liver is the first contact site for microbial components, endogenous, and exogenous toxins present in portal blood, being also responsible for initial immune response^32^.

Three proteins most abundant in LFE group were associated with higher body fat content in humans and mice and other studies also reported higher fat deposition in less efficient animals^23,33–35^. One of these proteins is *Ketohexokinase* (KHK) (co-expressed in the turquoise module) related with carbohydrate metabolism and the suppression of KHK gene in the liver of mice caused a reduction of enzymes of fatty acids synthesis^36^. Also related to carbohydrate metabolism, *Malate dehydrogenase*, *mitochondrial* (MDH2) protein (co-expressed in the red module) is a key enzyme in the citric acid cycle and its reduction in obese mice has been associated with body weight loss^37^. The other protein more abundant in LFE group (also in the red module) is the *Hydroxysteroid dehydrogenase-like protein 2* (HSDL2) which has a domain of *sterol carrier protein 2* (SCP2) that is involved in the transport and metabolism of lipids, suggesting participation on these pathways^38–41^. Olivieri *et al*.^12^ identified HSDL2 gene as a candidate in a genomic region (BTA8) associated with FE in Nellore cattle. The BTA8 region was also related to FE in Nellore cattle by Santana *et al*.^15^ and taurine animals by Lu *et al*.^42^, and several QTLs associated with RFI, feed intake and body weight gain were detected in this region by Seabury *et al*.^43^.

Pathways and processes related to energy metabolism were overrepresented for LFE group. *Glycolysis/gluconeogenesis* was enriched for the turquoise module and the DAPs *Alcohol dehydrogenases* (ADH), *Triosephosphate isomerase* (TPI1) and *Fructose-biphosphate aldolase B* (ALDOB) were more abundant in LFE group. Studies have shown that macrophage activation stimulated by LPS promotes an increase in glucose uptake and glycolysis, giving preference to this pathway to supply the energy demand of pro-inflammatory processes^44^. *Fatty acids degradation* was also overrepresented for turquoise module, and this is an important energy production pathway, especially for tissues that have a high metabolic demand, such as the liver, the main organ for homeostasis regulation in mammals^45^. Fatty acids are released from adipose tissue and absorbed by hepatocytes where they will be oxidized to ATP production, which will act as energy source or converted into lipid molecules and derivatives^46^.

ADHs and ALDHs are part of the Cyt P450, one of the most relevant complexes involved in the detoxification processes in mammals, whose pathways have been enriched for DAPs in LFE, and also for two co-expressed networks (turquoise and red modules). Other four proteins most abundant in LFE group and co-expressed in turquoise module are also related to Cyt P450: *Dimethylaniline monooxygenase [N-oxide-forming] 3* (FMO3), *Uncharacterized protein* (LOC540707), G*lutathione S transferase* (GSTM1/GSTM2) and *Sulfotransferase 1A1* (SULT1A1). Metabolism of drugs and xenobiotic by Cyt P450 has already been associated with FE in cattle^18,22,47–49^. In animals, along with the mitochondrial respiration processes, the metabolism of xenobiotic and inflammatory processes also produce oxidizing agents that contribute to formation of reactive oxygen species (ROS)^50^. ROS are formed both in normal physiological conditions and in the presence of stressors and oxidative stress^51,52^. As a consequence, antioxidant proteins were found more abundant in LFE animals and were found co-expressed in the turquoise module, such as *10-formyltetrahydrofolate dehydrogenase* (ALDH1L1), *Fumarylacetoacetate* (FAH), *Peroxirredoxin 2* (PRDX2), *Prostaglandin reductase 1* (PTGR1). Terms associated with oxidation-reduction were enriched for DAPs and presented mainly proteins more expressed in the LFE group. Alexandre *et al*.^16^ also observed enrichment of this process in a co-expression network negatively associated with FE, while Tizioto *et al*.^21^ suggested an increase in oxidative metabolism in less feed efficient Nellore. Oxidative stress has also been associated with low FE in other species of animal production such as poultry, pigs and other livestock species^53–56^.

Among the 19 more abundant proteins in HFE group, 12 were co-expressed in the brown module, a network associated with HFE. These proteins were mainly related to the processing, localization and transport of proteins associated with the endoplasmic reticulum (ER), amino acid metabolism and structural components. ER is an organelle that controls intracellular Ca^2+^ homeostasis, lipid synthesis and protein folding, which is highly sensitive to environmental changes, including redox state and presence of pathogens or inflammatory stimuli, resulting in accumulation of unfolded proteins or wrongly enveloped, which culminates in ER stress^57,58^. Greater abundance of proteins involved in prevention and correction of assembly and protein folding disorders in the HFE group may suggest that these animals are also undergoing oxidative stress, but they present higher capacity to adapt for this state, demanding less energy expenditure.

Based on the results obtained with the proteomic approach, in both differential expression and co-expression network analyzes, we are able to suggest the main metabolic pathways related to feed efficiency in the liver of beef cattle as: the microbial load from the gut; the biosynthesis of fatty acids, amino acids and vitamins; glycolysis/gluconeogenesis; cytochrome p450 xenobiotic metabolism and; antigen processing and presentation. We proposed that LFE animals present alterations in hepatic lipid metabolism and increased bacterial load where these changes lead to cellular homeostatic imbalance, culminating in oxidative stress. Thus, these animals present greater energy expenditure to maintain organism homeostasis, using the energy that could have been partitioned for body weight gain. Even though the proteome is dynamic and characterizes the tissue evaluated at the moment of sampling, the agreement between the results obtained in this approach with the transcriptomic data obtained by Alexandre *et al*.^16^, confirms that the proteomic analysis can help in the search for biomarkers for FE which can assist in herd feeding management and animal breeding.

## Conclusion

The findings presented here confirmed the altered hepatic metabolism and pronounced hepatic inflammation in LFE animals supporting that the increased bacterial load is at least in part responsible for the hepatic lesions and inflammation in LFE animals.

## Methods

### Bovine liver samples

This work followed an earlier study on FE in Nellore cattle of our group and detailed information about experimental design, animal management, and data collection can be found in Alexandre *et al*.^16^. Briefly, 98 young male Nellore bulls were maintained in the feedlot for 70 days feeding trial with a total mixed ration (including dry corn grain, corn silage, soybean, citrus pulp pellets, urea, calcareous, mineral salt and potassium chloride) offered ad libitum. These animals were classified for two FE traits: residual feed intake (RFI) and residual intake and body weight gain (RIG). RFI was obtained in accordance with Koch *et al*.^59^, as the difference between the observed and expected dry matter intake and RIG was calculated by the difference between residual body weight gain^59^ and RFI, as proposed by Berry and Crowley^60^. After the experiment, all animals were slaughter for consumption. At this moment, liver samples were collected, frozen in liquid nitrogen and stored at −80 °C until further analyses. The experimental protocols were approved by the Institutional Animal Care and Use Committee of Faculty of Food Engineering and Animal Sciences (CEUA/FZEA), University of São Paulo (USP) under number 14.1.636.74.1. All the experimental protocols were performed in accordance with CEUA/FZEA-USP guidelines and regulations.

### Protein extraction

Protein extraction was performed according to Xu *et al*.^61^ with minor modifications to six samples of each group. Briefly, frozen samples were minced and homogenized individually on ice in buffer containing 8 M urea, 65 mM DTT, 4% CHAPS and protease inhibitor (50 µl/g of sample, GE Healthcare, Little Chalfont, United Kingdom) and incubated on ice three hours under stirring. Then the samples were centrifuged at 4 °C for 30 min at 10,000 × g, the supernatant was carefully collected, and the proteins were precipitated in ice-cold acetone. The pellet was solubilized in buffer containing 8 M urea in 100 mM ammonium bicarbonate and stored at −80 °C until use.

### Mass spectrometry of protein samples

Protein samples were digested with trypsin and concentrated with the microcolumn *ZipTip*® C18 (Merck Millipore, Billerica, Massachusetts, USA). Mass spectrometry analysis was performed in the BIOMASS Core Facility for Scientific Research of University of São Paulo, Brazil and was carried out on an LTQ-Orbitrap Velos ETD (Thermo Scientific, Waltham, Massachusetts, USA) coupled with Easy NanoLC II (Thermo Scientific, Waltham, Massachusetts, USA). The peptides were separated on a C18 reverse phase column on a 90 min gradient. The analyses were carried out using higher energy collisional dissociation (HCD) for mass fragmentation and data-dependent acquisition (DDA) mode. The samples were analyzed in two technical replicates that were combined to perform the database search.

The acquired data were analyzed with MaxQuant software version 1.5.8.3^62,63^ for proteins identification searched against the *Bos taurus* (48,738 entries) and *Bos indicus* (1,252 entries) databases obtained from UniProt (versions from January 2018). The parameters used were: trypsin specificity; two missed cleavages; carbamidomethylation of cysteine as a fixed modification, methionine oxidation, protein amino-terminal acetylation, and deamidation (NQ) as variable modifications; initial precursor (MS) mass tolerance was 20 ppm in the first search and 6 ppm in the main search, and fragment (MS/MS) mass deviation of 20 ppm; peptide and protein false discovery rate (FDR) were both set at 1%. The “match-between-runs” algorithm was used with a match time window of 0.7 min and alignment time search space of 20 min. Label-free quantitation (LFQ) of identified proteins was performed with a minimum ratio count of 1^64–66^, and considered razor plus unique peptides for protein level quantitation.

### Statistical analysis

Statistical analysis was performed with the LFQ intensity values in Perseus software version 1.6.0.7^67^. Prior to the analyzes the contaminant proteins and matches to the reverse database were removed. Only proteins occurring in at least 50% of the biological replicates of both experimental groups were maintained and which have been identified with at least one unique peptide. The normalized LFQ intensity values were transformed by log2 and missing values were replaced by the random number drawn from a normal distribution. Student’s t-test was used for comparison between groups and proteins with a p-value < 0.05 were considered differentially abundant proteins (DAPs). Hierarchical clustering analysis was performed with DAPs after Z-score normalization.

### Bioinformatic analysis of differentially abundant proteins

The protein-protein interaction and Kyoto Encyclopedia of Genes and Genomes (KEGG) pathways enrichment analysis were performed with String software version 10.5^68^. The interaction network of DAPs was constructed using *B*. *taurus* interactions map and the connection definitions representing the type of evidence of interaction and minimum confidence score required of 0.40. KEGG pathways were considered enriched with p-value < 0.05, corrected to FDR with Benjamini-Hochberg method (p-adjust < 0.05). DAPs were also subjected to the functional categorization of Gene Ontology (GO) terms with the Analysis Toolkit and Database for Agricultural Community software (agriGO)^69^, applying the Hypergeometric statistical test with correction by the Bonferroni method, considering p-value < 0.05 as significant (p-adjust < 0.05), at least five proteins in each term, and the *B*. *taurus* genome as background. The GO terms were analyzed with Reduce + Visualize Gene Ontology (Revigo) software^70^ to remove redundant terms, adopting the SimRel semantic similarity measure and low threshold (0.50) with the other default parameters.

### Co-expression network analysis

All the identified proteins were subjected to co-expression analysis by package Weighted Gene Co-expression Network Analysis (WGCNA)^71,72^ available in software environment R. LFQ intensity values log2 transformed and imputed were used for this analysis. A Pearson correlation matrix was constructed, where the correlation signal was preserved (“*signed network*”), which was later converted into the weighted adjacency matrix, adjusting the correlations with a power β of 17, obtaining the free-scale topology with R^2^ = 0.85 and modules with a minimum size of 15 proteins. The modules were correlated to the FE traits (RIG and RFI) and correlations with p-value < 0.10 were considered significant. The names of the network modules (colors) were chosen by the software with no meaning regarding its function.

The proteins belonging to these modules with module membership (MM) values greater than 0.60 and p-value < 0.05 were visualized with Cytoscape version 3.6.0^73^. The enrichment of KEGG pathways was also performed with the String version 10.5 as previously described for DAPs. This analysis was performed for the significant modules using only proteins with MM ≥ 0.60 and p-value < 0.05 and MM < 0.60 with other modules.

### Serum endotoxin quantification

On the last day of the feeding trial (day 70), blood was collected from the jugular vein of all animals in vacuum tubes containing clot activator. The tubes were centrifuged (4 °C, 3500 rpm) for 15 minutes to separate the serum. Serum aliquots were stored in −80 °C freezer until analysis. Serum endotoxin dosage of 10 extreme animals (more animals than the proteome analysis but still high or low extreme animals for FE) of each FE group (HFE and LFE) was performed by using the Kinetic-QCL Kinetic Chromogenic LAL Assay (Lonza, 50-650U). For this purpose, the samples were preheated at 70 °C for 15 minutes and used pure or diluted 1:1 or 1:4 in order to dilute the endotoxins to reach the detection range of the kit for absolute quantification. One sample presented no absolute quantification, being indicated as <0.01 EU/ml and the value of 0.01 EU/ml was assigned to it for the statistical test. In addition, two samples presented values ≥2.5 SD from the mean of all samples and were withdraw from the analysis. High and low FE groups were tested by Student’s t-test.

## Supplementary information

Figure S1

Suplementary tables

## Data Availability

The datasets generated during and/or analyzed during the current study are available from the corresponding author on reasonable request.
