# Supplementary material for: Liver proteomics unravel the metabolic pathways related to Feed Efficiency in beef cattle
Source: Sci Rep. 2019 Mar 29;9:5364. doi: 10.1038/s41598-019-41813-x (PMC6441086; doi:10.1038/s41598-019-41813-x)
Supplement: Supplementary file 1 — Figure S1 [file 41598_2019_41813_MOESM1_ESM.pdf]

Liver proteomics unravel the metabolic pathways related to Feed Efficiency in beef cattle

Leydiana D. Fonseca[1], Joanir P. Eler[1], Mikaele A. Pereira[2], Alessandra F. Rosa[1], Pâmela A. Alexandre[1], Cristina T. Moncau[3], Fernanda Salvato[4], Lívia Rosa-Fernandes[5], Giuseppe Palmisano[5], José B. S. Ferraz[1], Heidge Fukumasu[1,*]

[1]Department of Veterinary Medicine, College of Animal Science and Food Engineering, University of São Paulo, Pirassununga, 13635-900, Brazil

[2] Department of Veterinary Medicine, Federal University of Sergipe, 49100-000, Brazil

[3] Department of Animal Science, University of Lavras, Lavras, 37200-000, Brazil

[4] Department of Plant and Microbial Biology, North Caroline State University, Raleigh, 27695, USA

[5] Department of Parasitology, Biomedical Sciences Institute, University of São Paulo, São Paulo, 05508-900, Brazil

*Correspondence and requests for materials should be addressed to H.F. (email: fukumasu@usp.br)

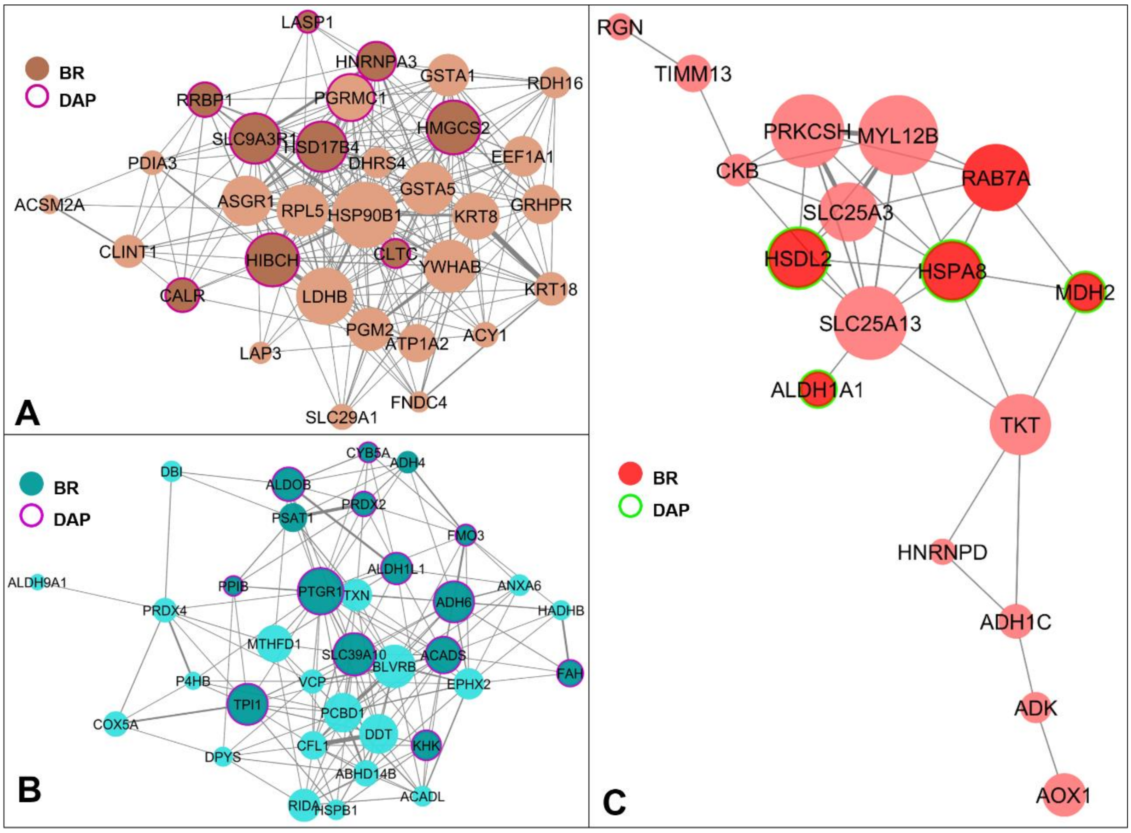

Figure S1 – Connectivity of hepatic proteins of Nellore cattle in co-expression networks related with feed efficiency. A: Connectivity in the brown module. B: Connectivity in the turquoise module. C: Connectivity in the red module. BR: biologically relevant proteins for feed efficiency, considering protein significance value > |0.60| (p-value < 0.05) for feed efficiency; DAP: differentially abundant proteins between Nellore cattle of high and low feed efficiency. Modules named by different colors represent co-expressed proteins network. Nodes represent the differentially abundant proteins that are identified with the coding gene symbol. Lines represent the connections between proteins.
